# Assessment of Blood Biophysical Properties Using Pressure Sensing with Micropump and Microfluidic Comparator

**DOI:** 10.3390/mi13030438

**Published:** 2022-03-13

**Authors:** Yang Jun Kang

**Affiliations:** Department of Mechanical Engineering, Chosun University, 309 Pilmun-daero, Dong-gu, Gwangju 61452, Korea; yjkang2011@chosun.ac.kr; Tel.: +82-62-230-7052

**Keywords:** biophysical property, micropump, microfluidic comparator, junction pressure, pressure-induced work, RBC aggregation, fluidic circuit model, blood viscosity

## Abstract

To identify the biophysical properties of blood samples consistently, macroscopic pumps have been used to maintain constant flow rates in a microfluidic comparator. In this study, the bulk-sized and expensive pump is replaced with a cheap and portable micropump. A specific reference fluid (i.e., glycerin solution [40%]) with a small volume of red blood cell (RBC) (i.e., 1% volume fraction) as fluid tracers is supplied into the microfluidic comparator. An averaged velocity (<*U_r_*>) obtained with micro-particle image velocimetry is converted into the flow rate of reference fluid (*Q_r_*) (i.e., *Q_r_* = *C_Q_* × *A_c_* × <*U_r_*>, *A_c_*: cross-sectional area, *C_Q_* = 1.156). Two control variables of the micropump (i.e., frequency: 400 Hz and volt: 150 au) are selected to guarantee a consistent flow rate (i.e., COV < 1%). Simultaneously, the blood sample is supplied into the microfluidic channel under specific flow patterns (i.e., constant, sinusoidal, and periodic on-off fashion). By monitoring the interface in the comparator as well as *Q_r_*, three biophysical properties (i.e., viscosity, junction pressure, and pressure-induced work) are obtained using analytical expressions derived with a discrete fluidic circuit model. According to the quantitative comparison results between the present method (i.e., micropump) and the previous method (i.e., syringe pump), the micropump provides consistent results when compared with the syringe pump. Thereafter, representative biophysical properties, including the RBC aggregation, are consistently obtained for specific blood samples prepared with dextran solutions ranging from 0 to 40 mg/mL. In conclusion, the present method could be considered as an effective method for quantifying the physical properties of blood samples, where the reference fluid is supplied with a cheap and portable micropump.

## 1. Introduction

Hemorheological disorders and diseases are related to alterations in blood samples, including those concerning plasma proteins, hematocrit (Hct), and the stiffness (or deformability) of cells and plasma membrane [1,2]. These alterations contribute to a reduction of mass transport and interrupt the blood flow in capillary vessels. To effectively detect changes in blood samples from a physical point of view, several rheological properties (i.e., viscosity [3,4,5,6], viscoelasticity [7], red blood cell (RBC) deformability [8,9,10,11,12,13], RBC aggregation [14,15,16,17], RBC sedimentation rate [18,19], and Hct [20]) have been measured for screening or diagnosing various diseases, including coronary heart disease, hypertension, diabetes [21], sickle cell anemia [22,23], and malaria. Plasma protein (i.e., fibrinogen) contributes to increasing RBC aggregation. It tends to reduce blood flow in venules or veins, as an elevated RBC aggregation leads to increased fluidic resistance (or blood viscosity). As the number of RBCs is much larger than the number of other cells or cell elements (i.e., white blood cells and platelets), most of the biophysical properties of blood samples have been determined by RBCs. Thus, most researchers have focused on RBCs and plasma protein [24].

Among the several biophysical properties of blood samples, blood viscosity is influenced by several factors, including the plasma protein, number of RBCs, deformation of RBCs, and plasma membrane. When blood samples flow in a microfluidic channel (i.e., with a higher shear rate), the blood viscosity is determined by RBC deformability, rather than the RBC aggregation. The hematocrit has a strong influence on blood viscosity. The flow rate contributes to changing the blood viscosity because it varies as a function of the shear rate (i.e., in a non-Newtonian fluid). Recently, microfluidic devices have been considered as promising tools for quantifying the biophysical properties of blood samples [1,25]. After placing the blood samples into a microfluidic channel, the blood viscosity is obtained by quantifying the fluidic resistance (i.e., pressure drop = fluidic resistance × flow rate) [26] under a pressure drop [4,5] or flow rate [3,27].

Among several methods for measuring blood viscosity [2], a microfluidic comparator (or a corresponding modification device) has been considered as providing a simple and convenient method. According to the working principle of a comparator [3,4], the flow rates of both fluids should be specified in advance and maintained consistently throughout experiments. Therefore, two highly precise pumps are required to maintain a constant flow rate. However, during blood delivery, RBC sedimentation occurs continuously in the driving syringe and/or in the long tubing. Even when the flow rate of the blood sample is set to a constant value with the syringe pump, the hematocrit of the blood sample tends to vary continuously [28]. The hemorheological behavior of blood flow changes over time [29,30].

Although a blood flow rate can be estimated consistently with micro-particle image velocimetry (PIV) [31], it is very difficult to obtain an accurate flow rate of a blood sample. The flow rate obtained with micro-PIV varies strongly with several factors, such as flow rate, hematocrit, diluent, RBC aggregation, and RBC deformability [13,14,29]. Whenever blood viscosity is to be monitored, tedious calibration procedures are required in advance using the same blood sample. To resolve a critical issue raised in the previous study, our group has measured junction pressure, rather than blood viscosity [32]. As both streams in the comparator have the same pressure drop, it is possible to get the junction pressure of the blood stream indirectly. In addition, the flow rate of the reference fluid should be set to a constant value. Without quantitative information on blood flows, the junction pressure of blood flow can be estimated from quantitative information of the reference fluid stream. This method does not require quantitative information on the blood flow (e.g., the blood viscosity and flow rate). Nevertheless, according to a previous study [32], a highly precise syringe pump is required to maintain a constant flow rate of the reference fluid.

In this study, a highly expensive and precise pump is replaced with a cheap and portable micropump. The micropump is adopted to improve portability and convenience when compared with the macroscopic pump. This study aims to assess the biophysical properties of blood (i.e., junction pressure and pressure-induced work) using a micropump and microfluidic comparator. However, several issues exist when adopting a micropump in this manner. The flow rate of the micropump varies depending on the junction pressure in the microfluidic comparator. Therefore, it is necessary to quantify the flow rate of the reference fluid continuously during experiments. As a simple method, a microparticle image velocimetry (PIV) technique is employed to quantify variations of the flow rate of the reference fluid over time. Here, a small volume fraction of RBCs as fluid tracers is added into the reference fluid. If 1× phosphate-buffered saline (PBS) is selected as the reference fluid, RBC sedimentation occurs because the density of the RBCs is higher than that of the 1× PBS (pH 7.4, Gibco, Life Technologies, South Korea) [10,33]. After a certain time, there are relatively few RBCs floating inside the reference fluid stream. It is impossible to obtain consistent velocity fields for the reference fluid over a sufficiently long time (approximately 1 h). Therefore, instead of 1× PBS, a glycerin solution (40%) is selected as the reference fluid to avoid RBC sedimentation [29].

Analytic expressions for the three properties of the blood sample (i.e., viscosity, junction pressure, and pressure-induced work) are derived using a discrete fluidic circuit model. The accuracy of the mathematical model is validated and compared with experimental results. The present method is then adopted to quantify the biophysical properties of blood samples. The results obtained from the present method (i.e., micropump) are compared quantitatively with the results obtained from the previous method (i.e., a syringe pump). Finally, the present method is employed to quantify the variations in biophysical properties (i.e., the junction pressure, pressure-induced work, and RBC aggregation) of blood samples with different concentrations of dextran solution, where the blood sample flows in a periodic on-off fashion.

When compared with the previous method, instead of a highly expensive and precise macroscopic pump, a cheap and portable micropump is adopted to supply reference fluid in the microfluidic comparator. In addition, a specific concentration of glycerin solution (40%) is selected as a reference fluid for maintaining consistent distributions of RBCs in the reference fluid stream during a long period of delivery (~1 h). The control variables of the micropump (i.e., frequency [*f*] = 400 Hz and volt [*v*] = 150 au) are optimized experimentally to minimize fluctuations in flow rate (i.e., COV < 1%). By monitoring the flow rate of the reference fluid and interface in the comparator, the present method has the ability to quantify the physical properties (i.e., junction pressure and pressure-induced work) of blood samples where the reference fluid is supplied with the micropump. Here, the flow rate of the blood sample is controlled at various profiles (i.e., constant flow rate, sinusoidal flow rate, and periodic on-off flow rate). According to the quantitative comparison studies, the micropump (i.e., present study) provides consistent results when compared with the syringe pump (i.e., previous study).

## 2. Materials and Methods

### 2.1. Design and Fabrication of Microfluidic Device, and Experimental Procedure

**Figure 1:**
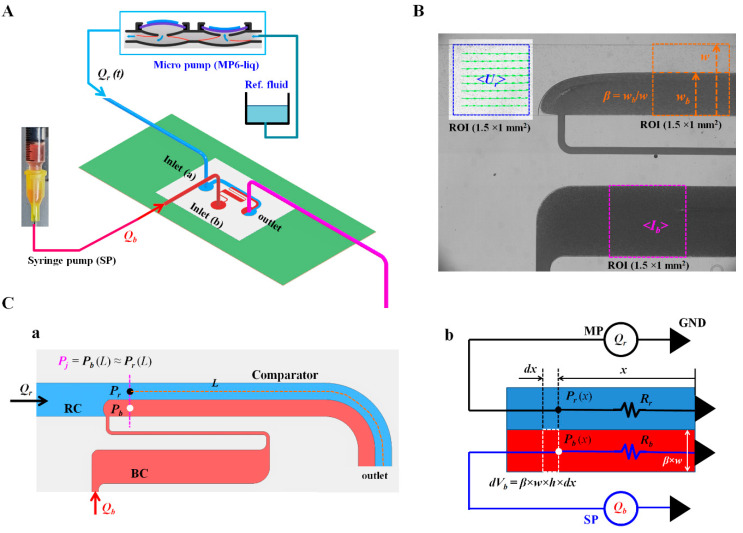
The proposed method for quantifying blood biophysical properties by analyzing blood flows in a microfluidic device. (**A**) Schematic diagram of the proposed method. The method employed a microfluidic device, micropump for supplying reference fluid, and syringe pump for delivering blood. (**B**) Quantification of three parameters (i.e., <*U_r_*>, <*I_b_*>, and *β*). Each parameter was arithmetically averaged over a specific region of interest (ROI) (1.5 × 1 mm^2^). (**C**) Analytical representation of the blood biophysical properties using a discrete fluidic circuit model. (**a**) Junction pressure of each fluid stream in the comparator with length (*L*). Both streams had the same pressure (i.e., *P_j_* = *P_r_*[*L*] = *R_b_*[*L*]). (**b**) Discrete fluidic circuit model of the comparator. For convenience, the comparator was simply modeled as two fluidic resistances (*R_r_*, *R_b_*) connected in parallel. Flow rates controlled by a micropump (MP) as well as a syringe pump (SP) were represented as *Q_r_* and *Q_b_* for reference fluid and blood sample, respectively. At distance (*x*) from the outlet, infinitesimal blood volume (*dV_b_*) was expressed as *dV_b_* (*x*) = *β* × *w* × *h* × *dx*. Pressure-induced work of the blood stream in the comparator (*W_b_*) was then derived as *W_b_* = 0.5 *P_r_* (*L*) × *V_b_* (*L*).

As shown in Figure 1A,C(a), the microfluidic device was comprised of a reference channel (RC), blood channel (BC), comparator, two inlets (a,b), and an outlet. The RC and comparator had the same channel width (*w*) of *w* = 1000 µm. The BC had three components connected in series (i.e., a narrow channel (*w* = 100 µm), big channel (*w* = 1000 µm), and narrow channel (*w* = 100 µm)). As the narrow channel contributed to avoiding invasion of reference fluid stream, the RBC aggregation of the blood samples within the BC was monitored effectively under an on-off blood flow. The depth (*h*) of all channels was fixed at 20 µm.

Based on microfabrication procedures (i.e., photolithography and soft lithography) reported in a previous study [34], a polydimethylsiloxane (PDMS, Sylgard 184, Dow Corning, Midland, MI, USA) slab was replicated from a 4-inch silicon wafer. Two inlets (a, b) and one outlet were punched using a biopsy punch (outer diameter = 0.75 mm). The PDMS slab and glass substrate were treated with oxygen plasma (CUTE-MPR; Femto Science Co., Hwaseong-si, Korea). They were subsequently bonded together.

The microfluidic device was placed on an inverted microscope (IX53; Olympus, Tokyo, Japan) with a 5× objective lens (NA = 0.15). The inlet and outlet ports of the micropump (MP6-liq, Bartels Mikrotechnik GmbH, Germany) were tightly fitted with two polyethylene tubings (length = 300 mm, inner diameter = 0.8 mm). One end of the tubing was connected to the buttonhole of a centrifuge tube (5 mL) which was filled with the reference fluid (glycerin solution [40%]). The other end of the tubing was fitted to an inlet (a). Two polyethylene tubes (length = 300 mm, inner diameter = 0.25 mm) were tightly connected to the inlet (b) and outlet, respectively.

To remove the air inside the channels and the non-specific bonding of plasma proteins, bovine serum albumin (BSA, 1 mg/mL) was supplied through the outlet. After 10 min elapsed, the channels were filled with 1× PBS after removing the BSA. A disposable syringe filled with the blood sample was connected to the end of the tubing connected to the inlet (b). The syringe was then placed in a syringe pump (NeMESYS; Cetoni GmbH, Korbußen, Germany). The flow rate of the reference fluid was changed immediately by setting the frequency and voltage of the micropump.

### 2.2. Image Acquisition and Processing for Quantifying Three Parameters

A high-speed camera (FASTCAM MINI, Photron, Tokyo, Japan) was used to capture microscopic images of blood flows. The frame rate of the camera was set to 2000 frames/s. Two microscopic images were captured sequentially at intervals of 0.2 s for a certain duration. All experiments were conducted at a constant temperature of 20 °C.

To quantify the biophysical properties of blood samples, three parameters were quantified by analyzing the microscopic images captured over time. First, because the flow rate of the reference fluid varies depending on the junction pressure, it was necessary to quantify the flow rate using micro-PIV [31]. A specific region of interest (ROI) (1.5 × 1 mm^2^) located upstream from the comparator was selected within the RC. The interrogation window was set to 64 pixels × 64 pixels. One pixel in the blood image equaled a 3.3-µm square. The window overlap was set to 50%. The velocity fields within the ROI were validated by using a local median filter. The average velocity (<*U_r_*>) was obtained by averaging the velocity fields distributed over the ROI. Second, to quantify the RBC aggregation of the blood samples, the image intensity of the blood flow was calculated within a specific ROI (1.5 × 1 mm^2^) within a large channel of the BC. Subsequently, the intensity of blood flow (<*I_b_*>) was obtained by averaging the intensity distributed over the ROI. To consider the pure contributions of the blood sample, the image intensity from no blood flow (i.e., the background image) was subtracted from <*I_b_*>. Finally, to quantify the junction pressure in the comparator, the interface between the blood stream and reference fluid stream was detected within a specific ROI of the comparator (1.5 × 1 mm^2^); this was located downstream from the RC. To effectively detect the interface, the grayscale image was converted into a binary image using a level-thresholding algorithm. The width of the bloodstream (*w_b_*) was obtained by averaging the interface as distributed over the ROI. The normalized interface of the bloodstream (*β*) was estimated as *β* = *w_b_*/*w*.

### 2.3. Quantification of Blood Biophysical Properties

Without a specific flow rate for the blood sample, it was impossible to obtain the blood viscosity or junction pressure in the comparator. However, based on the fact that both streams had the same pressure in the comparator, the junction pressure of the blood stream could be estimated indirectly using the junction pressure of the reference fluid stream. Namely, the flow rate of the reference fluid was quantified using the micro-PIV technique. The viscosity of the reference fluid was determined in advance. It was necessary to construct a simple mathematical model to estimate the junction pressure in the comparator. The junction pressure in the comparator was estimated analytically based on the discrete fluidic circuit model. Additionally, the pressure-induced work was derived by multiplying the pressure with the blood volume.

As shown in Figure 1C(a), the reference fluid and blood sample flowed into the RC and BC, respectively. These streams were joined and separated into the comparator. The flow rate of each fluid was modeled as *Q_r_* and *Q_b_,* respectively. As the reference fluid was delivered with a micropump, *Q_r_* varied depending on the junction pressure. Based on the working principle, quantitative information on *Q_b_* was not required to estimate the junction pressure. Thus, *Q_b_* did not need to be specified. The width of each stream was assumed to be (1 − *β*) × *w* for the reference fluid stream and *β* × *w* for the blood stream. The length of the comparator was defined as *L*. *L* varied from 8.7 mm to 9.4 mm. Specifically, both streams had the same junction pressure (i.e., *P_j_* = *P_r_*(*L*) = *P_b_*(*L*)) at distance (*L*). Here, the subscripts (*j*, *r*, and *b*) represent the junction, reference fluid stream, and blood stream, respectively.

As shown in Figure 1C(b), to derive an analytical formula for the junction pressure, the comparator was modeled using discrete fluidic circuit elements (i.e., flow rates and fluidic resistances). For convenience, the comparator was divided into two streams (i.e., the reference fluid stream and bloodstream) with a virtual wall. The comparator was modeled as two fluidic resistances (*R_r_* and *R_b_*) connected in parallel. At distance (*x*) from the outlet, the junction pressures of both streams were expressed as *P_r_*(*x*) for the reference stream and *P_b_*(*x*) for the bloodstream. According to the Poiseuille law (i.e., pressure drop = fluidic resistance × flow rate) for each stream, *P_r_*(*x*) and *P_b_*(*x*) were given analytically by Equations (1) and (2), respectively, as follows:

*P_r_*(*x*) = *Q_r_* × *R_r_* (1)

*P_b_*(*x*) = *Q_b_* × *R_b_*/*C_R_* (2)

Regarding the difference between the real boundary and modeling boundary, a correction factor (*C_R_*) obtained by experimental procedure or numerical simulation was inserted into the expression of *P_b_*(*x*), based on a previous study [35]. The fluidic resistance of each stream (i.e., the width of the reference fluid stream = [1 − *β*] × *w*, and width of the blood stream = *β* × *w*) [32] were derived analytically using Equations (3) and (4), respectively, as follows:

(3) $$R_{r}=\frac{12\times \mu_{r}\times x\;}{\left(1-\beta \right)\times w\times h^{3}}$$

(4) $$R_{b}=\frac{12\times \mu_{b}\times x\;}{\beta \times w\times h^{3}}$$

Here, *µ_r_* and *µ_b_* represent the viscosities of the reference fluid and blood sample, respectively. By inserting Equations (3) and (4) into Equations (1) and (2), *P_r_*(*x*) and *P_b_*(*x*) were then approximated as follows:

(5) $$P_{r}\left(x\right)=\left(\frac{12\times \mu_{r}\times x\;}{\left(1-\beta \right)\times w\times h^{3}}\right)\times Q_{r}$$

(6) $$P_{b}\left(x\right)=\left(\frac{12\times \mu_{b}\times x\;}{C_{R}\times \beta \times w\times h^{3}}\right)\times Q_{b}$$

According to Equation (6), *P_b_* was expressed as two specific parameters (*µ_b_* and *Q_b_*). *Q_r_* was controlled by the micropump. It was quantified using the micro-PIV technique. The interface (*β*) in the comparator was obtained by analyzing the microscopic images. Based on the same junction pressure in the comparator (i.e., *P_r_*[*x*] = *P_b_*[*x*]), the junction pressure of the blood stream was estimated indirectly using Equation (5) (i.e., *P_j_* = *P_r_*[*x*]). The same pressure condition in the comparator provided a relationship as follows:

(7) $$\left(\frac{12\times \mu_{r}\times x\;}{\left(1-\beta \right)\times w\times h^{3}}\right)\times Q_{r}=\left(\frac{12\times \mu_{b}\times x\;}{C_{R}\times \beta \times w\times h^{3}}\right)\times Q_{b}$$

According to Equation (7), the correction factor (*C_R_*) was derived as follows:

(8) $$C_{R}=\left(\frac{1-\beta }{\beta }\right)\times \left(\frac{Q_{b}}{Q_{r}}\right)\times \left(\frac{\mu_{b}}{\mu_{r}}\right)$$

**Figure A1:**
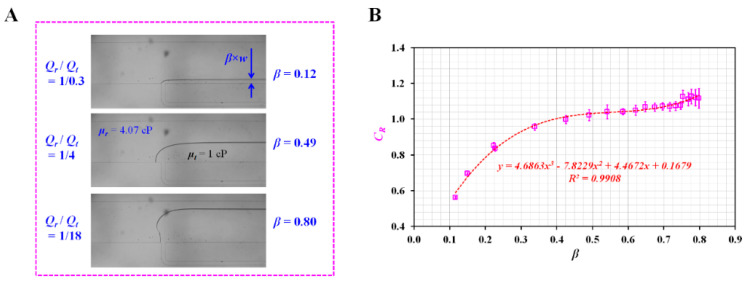
Experimental measurement of correction factor (*C_R_*). (**A**) Microscopic images showing the interfacial location (*β*) with respect to flow rate ratio of two fluids (*Q_r_*/*Q_t_*). The corresponding *β* for flow rate ratio (*Q_r_*/*Q_t_*) was obtained as *β* = 0.12 for *Q_r_*/*Q_t_* = 1/0.3, *β* = 0.49 for *Q_r_*/*Q_t_* = 1/4, and *β* = 0.8 for *Q_r_*/*Q_t_* = 1/18. (**B**) Variations of correction factor (*C_R_*) with respect to *β*. Linear regression analysis showed that *C_R_* was expressed as *C_R_* = 4.6863*β*^3^ − 7.8229*β*^2^ + 4.4672*β* + 0.1679 (*R*^2^ = 0.9908).

Equation (8) indicates that *C_R_* varies with respect to the interface (*β*) if the flow rate ratio (*Q_b_*/*Q_r_*) and viscosity ratio (*µ_b_*/*µ_r_*) are fixed. As shown in Figure A1, an experiment was conducted to determine the correction factor. Instead of the blood sample, 1× PBS was injected into the blood channel. Two syringe pumps were used to independently set the flow rates of the two fluids. The flow rate of the reference fluid was set to 1 mL/h (*Q_r_* = 1 mL/h). The flow rate of the test fluid was gradually increased from 1 mL/h to 18 mL/h. The viscosities of the reference and test fluids were *µ_r_* = 4. 08 cP and *µ_t_* = 1 cP, respectively [36]. Figure A1A shows microscopic images of the interfacial location (*β*) with respect to the flow rate ratio of the two fluids (*Q_r_*/*Q_t_*). The corresponding *β* for flow rate ratio (*Q_r_*/*Q_t_*) was obtained as *β* = 0.12 for *Q_r_*/*Q_t_ =* 1/0.3*, β* = 0.49 for *Q_r_*/*Q_t_ =* 1/4*,* and *β* = 0.8 for *Q_r_*/*Q_t_* = 1/18. As shown in Figure A1B, *C_R_* was then obtained with respect to *β* by inputting the given data (i.e., *β*, *Q_r_*/*Q_t_*, and *µ_t_*/*µ_r_*) into Equation (8). By conducting a linear regression analysis, *C_R_* was obtained as a function of *β* (i.e., *C_R_* = 4.6863*β*^3^ − 7.8229*β*^2^ + 4.4672*β* + 0.1679, *R*^2^ = 0.9908). The expression of the correction factor (*C_R_*) was only employed to quantify the blood viscosity when the flow rate of the blood sample was specified.

Next, the pressure-induced work of the blood stream in the comparator was quantified by multiplying the junction pressure by the blood volume. At distance (*x*) from the outlet, the infinitesimal blood volume (*dV_b_*) was estimated as *dV_b_*(*x*)= *β* × *w* × *h* × *dx*. The infinitesimal work (*dW_b_*) was expressed as follows:

(9) $$dW_{b}=P_{j}\times dV_{b}\left(x\right)\;=\left(\frac{12\;\mu_{r\;}x}{\left(1-\beta \right)wh^{3}}\times Q_{r}\right)\times \left(\beta \;w\;h\;dx\right)$$

By integrating Equation (9) from *x* = 0 to *x* = *L* (i.e., $W_{b}=\int_{0}^{L}dW_{b})$, the pressure-induced work of the blood stream in the comparator (*W_b_*) was derived as follows:

(10) $$W_{b}=\left(\frac{6\;\mu_{r\;}L}{\left(1-\beta \right)wh^{3}}\times Q_{r}\right)\times \left(\beta \;w\;h\;L\right)\;=0.5\;P_{r\;}\left(L\right)\times V_{b}\left(L\right)$$

In Equation (10), the blood volume in the comparator was given by *V_b_*(*L*) = *β* × w × *h* × *L*.

### 2.4. Blood Sample Preparation

This study was approved by the ethical committee of Chosun University under Reference code (2-1041055-AB-N-01-2021-80). The concentrated RBCs were ordered from the Gwangju–Chonnam Blood Bank (Gwangju, Korea). The samples were kept in a refrigerator before the experiments. The normal RBCs were collected by washing the concentrated RBCs in 1× PBS and removing debris. The blood samples (Hct = 50%) were prepared by suspending the normal RBCs in dextran solutions or 1× PBS. Four dextran solutions (*C_dex_* = 0, 10, 20, 30, and 40 mg/mL) were prepared by dissolving dextran powder (*Leuconostoc* spp., MW = 450–650 kDa; Sigma-Aldrich, Burlington, MA, USA) in 1× PBS. Here, *C_dex_* = 0 represents the 1× PBS.

## 3. Results and Discussion

### 3.1. Validation of Flow Rate of Reference Fluid Using Micro-PIV

When the micropump was used to supply the reference fluid, the flow rate varied depending on the junction pressure in the comparator. For this reason, it was necessary to quantify the variations in the flow rate under the junction pressure in the comparator. Micro-PIV was used to evaluate the velocity fields of the reference fluid in the RC. To quantify the velocity fields with consistency, fluid tracers were uniformly distributed into the reference fluid stream during the experiments. Therefore, the selection of the reference fluid was considered a critical issue. In addition, as the flow rate quantified with micro-PIV did not agree with the given flow rate (i.e., the flow rate of the syringe pump), it was necessary to determine the calibration procedure in advance.

**Figure 2:**
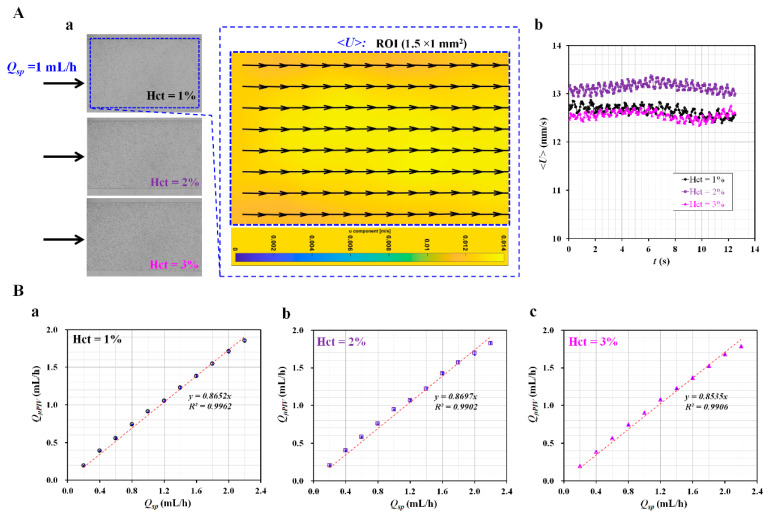
Validation of flow rate of reference fluid stream with micro-particle image velocimetry (PIV). (**A**) Microscopic images of reference fluid and averaged velocity (<*U*>) obtained with micro-PIV with respect to red blood cell (RBC) volume. (**a**) Three microscopic images with respect to RBC volume (hematocrit (Hct)). The right panel shows velocity fields of reference fluid (Hct = 1%). (**b**) Temporal variations of <*U*> with respect to RBC volume (Hct). (**B**) Linear relation between flow rate of reference fluid obtained with micro-PIV and flow rate set by syringe pump with respect to RBC volume (Hct) ((**a**) Hct = 1%, (**b**) Hct = 2%, and (**c**) Hct = 3%). The corresponding linear slope for each RBC volume faction was obtained as 0.8652 for Hct = 1%, 0.8697 for Hct = 2%, and 0.8535 for Hct = 3%.

**Figure A2:**
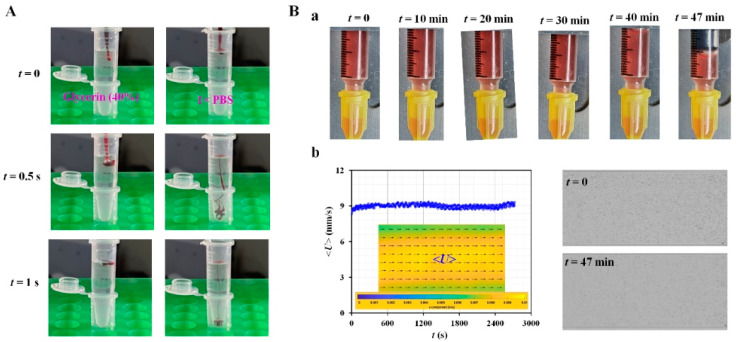
Validation of RBC sedimentation in reference fluid. (**A**) Sedimentation test of RBCs suspended in two different fluids (i.e., glycerin solution (40%) and 1× PBS) with an elapse of time (*t*) (*t* = 0, 0.5 and 1 s). (**B**) Validation of RBC sedimentation in glycerin solution (40%). (**a**) Snapshots for evaluating RBC sedimentation in a driving syringe over time (*t*) (*t* = 0, 10, 20, 30, 40, and 47 min). (**b**) Temporal variation of averaged velocity (<*U*>) over time. Inset shows the velocity fields of the reference fluid. Right panel shows microscopic images captured at an initial time (*t* = 0) and final time (*t* = 47 min).

First, a small volume fraction of RBCs was inserted into the reference fluid instead of polymer particles (i.e., diameter = 0.5–1 µm). The RBCs in the reference fluid were used as fluid tracers. Depending on the density difference between the RBCs and the reference fluid, the RBCs could potentially disappear in the reference fluid stream, owing to RBC sedimentation. To avoid RBC disappearance from the reference fluid stream, the density of the reference fluid needed to be larger than that of the RBCs (i.e., *ρ_RBC_* = 1.085–1.122 g/mL) [16,33]. Instead of 1× PBS (*ρ_PBS_* = 1 g/mL), a glycerin solution (40%) (*ρ_Glycerin_* =1.1145 g/mL) was selected as the reference fluid [37]. Figure A2A shows the sedimentation of RBCs as suspended in the two different fluids (i.e., glycerin solution [40%] and 1× PBS) with an elapsed time (*t*) (*t* = 0, 0.5, and 1 s). When RBCs were suspended into 1× PBS, the RBCs sank and became stacked on the bottom of the tube. In contrast, the RBCs floated and remained above the top surface of the glycerin solution (40%). Thereafter, the RBC sedimentation inside the glycerin solution (40%) was validated within the driving syringe and microfluidic channel. After the reference fluid was prepared by adding RBCs (~10 µL) to the glycerin solution (1 mL) (Hct = 1%), it was suctioned into the driving syringe. After placing it into a syringe pump, the flow rate of the reference fluid was set to 1 mL/h. Figure A2B(a) shows snapshots captured to evaluate the RBC sedimentation in the driving syringe at specific times (*t*) (*t* = 0, 10, 20, 30, 40, and 47 min). The images indicate that RBC sedimentation in the reference fluid does not occur for a long delivery time. Figure A2B(b) shows the temporal variation in the averaged velocity (<*U*>) over time. The inset shows the velocity field of the reference fluid. The right panel shows microscopic images captured at the initial (*t* = 0) and final times (*t* = 47 min). The results show that the RBCs in the reference fluid are distributed uniformly in the microfluidic channel, even after an elapsed time of 47 min. After the glycerin solution (40%) was selected as the reference fluid, the next step was to determine the volume of RBCs to be added to the reference fluid. In addition, the contribution of the added RBC volume (i.e., Hct: RBC volume fraction in relation to the reference fluid volume) was evaluated by measuring the flow rate of the reference fluid with micro-PIV, specifically, under a constant flow rate using a syringe pump. The reference fluid was prepared by adding RBC volumes (10, 20, and 30 µL) to 1× PBS (i.e., 1000 µL). The Hct values of the reference fluid were calculated as Hct = 1%, 2%, and 3%. Figure 2A(a) shows three microscopic images for Hct = 1%, 2%, and 3%. The flow rate of the reference fluid was set at 1 mL/h using a syringe pump (*Q_sp_* = 1 mL/h). According to the microscopic images, the number of RBCs inside the reference fluid stream increases significantly at higher values of Hct.

The reference fluid flowed from the left to the right side of the RC. The right panel shows the velocity field of the reference fluid for the RBC volume fraction (Hct = 1%). The velocity fields show uniform distributions within the specific ROI (1.5 × 1 mm^2^) of the RC. Figure 2A(b) shows the temporal variations of <*U*> with respect to the Hct. The corresponding averaged velocity (<*U*>) of each Hct is <*U*> = 12.65 ± 0.1 mm/s (Hct = 1%), <*U*> = 13.14 ± 0.09 mm/s (Hct = 2%), and <*U*> = 12.56 ± 0.08 mm/s (Hct = 3%). The averaged velocity of the reference fluid did not exhibit a substantial trend (i.e., increasing or decreasing) with respect to the Hct. Based on the analytical formula of the depth-of-correlation (DOC) [38], the DOC was estimated as 151.6 µm for the microscopic imaging system. As the DOC was much higher than the channel depth, micro-PIV quantified velocity fields were averaged along the depth direction. The flow rate of the reference fluid (*Q_µPIV_*) was obtained by multiplying the averaged velocity (<*U*>) by the cross-sectional area (*A_c_*) (i.e., *Q_µPIV_* = <*U*> × *A_c_*). To evaluate the contribution of the RBC volume faction to *Q_µPIV_*, the flow rate of the syringe pump (*Q_sp_*) was varied from *Q_sp_* = 0.2 mL/h to *Q_sp_* = 2.2 mL/h at intervals of 0.2 mL/h. The relationship between *Q_µPIV_* and *Q_sp_* was determined by drawing an X-Y plot (i.e., X-axis: *Q_sp_* and Y-axis: *Q_µPIV_*) and conducting a linear regression analysis. As shown in Figure 2B, three X-Y plots were drawn with respect to the RBC volume fraction (Hct) (Hct = 1%, 2%, and 3%). The plots indicate that *Q_µPIV_* is linearly proportional to *Q_sp_* with respect to Hct. Furthermore, according to linear regression analysis, the regression coefficient has an extremely high value of *R*^2^ = 0.9902–0.9962. The corresponding linear slope of the RBC volume fraction is 0.8652 for Hct = 1%, 0.8697 for Hct = 2%, and 0.8535 for Hct = 3%. Within 3% Hct, the slope (i.e., *Q_µPIV_*/*Q_sp_*) ranges from 0.8535 to 0.8697. Because the variation in the slope is much smaller within the RBC volume fraction (Hct = 3%), the RBC volume fraction does not have a strong influence on *Q_µPIV_*. However, with respect to a higher Hct value (Hct = 30–50%), *Q_µPIV_* has a nonlinear relationship with respect to *Q_sp_* [29]. Next, *Q_µPIV_* was best fitted as a polynomial function of *Q_sp_*. When the Hct increased significantly, the RBC-to-RBC interactions, deformability, and RBC aggregation influenced the varying velocity fields as obtained with micro-PIV [20,39]. For convenience, the RBC volume fraction (Hct) of the reference fluid was fixed at Hct = 1%. In addition, the linear slope was used as a correction coefficient to correct the flow rate obtained using micro-PIV.

### 3.2. Selection of Control Variables for Consistent Flow Rate with Micropump

Following the selection of the reference fluid as discussed in the previous section, it was necessary to select the operating conditions for the micropump. The micropump was controlled by two variables (i.e., the frequency and voltage). According to the working principle of a micropump, pulsatile flows were generated at higher flow rates (or near resonance frequency) [40,41,42]. As a consistent flow rate of the reference fluid was preferable during the experiments, it was necessary to select the frequency and voltage to minimize the pulsatile flow of the reference fluid.

**Figure 3:**
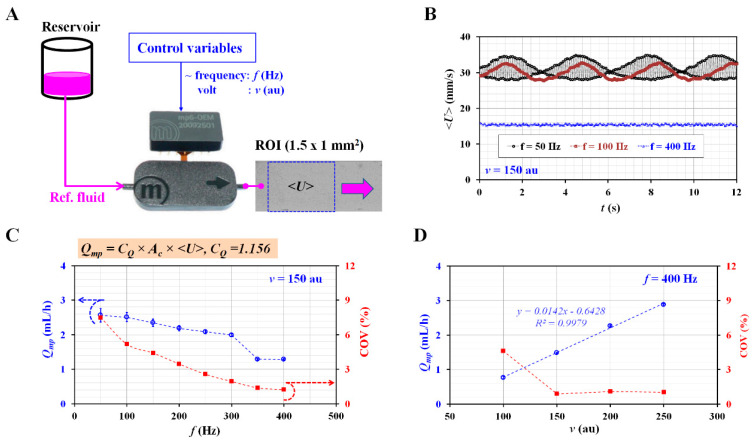
Quantitative evaluation of flow rate of reference fluid varied by setting control variables (i.e., frequency and voltage) of a controller connected to a micropump. (**A**) Schematic diagram of the experimental setup. (**B**) Temporal variations of <*U*> with respect to *f* = 50, 100, and 400 Hz. Here, the voltage was set to *v* = 150 au. (**C**) Variations of *Q_mp_* and COV with respect to *f* = 50, 100, 150, 200, 250, 300, 350, and 400 Hz. Here, the voltage was set to *v* = 150 au. The flow rate of the micropump (*Q_mp_*) was obtained as *Q_mp_* = *C_Q_* × *A_c_* × <*U*>. The corrector factor (*C_Q_*) was given as 1.156. (**D**) Variations of *Q_mp_* and coefficient of variance (COV) with respect to *v* = 100, 150, 200, and 250 au. Here, the frequency was set to *f* = 400 Hz.

Figure 3A shows a schematic of the experimental setup for evaluating the flow rate of the reference fluid controlled with the micropump. The micropump was installed between the reservoir and the microfluidic device. The reservoir was filled with a reference fluid with a specific RBC volume fraction (Hct = 1%). The controller was connected to the micropump. The flow rate (or velocity) of the reference fluid was varied by adjusting the control variables (i.e., frequency *f* and voltage *v*) sent to the controller. The averaged velocity of the reference fluid (<*U*>) was obtained by averaging the velocity fields distributed within a specific ROI (1.5 × 1 mm^2^). As shown in Figure 3B, the temporal variations in <*U*> were obtained by varying the frequency (*f*) (*f* = 50, 100, and 400 Hz). Here, the voltage was set to *v* = 150 au. When *f* was set to 50 Hz, <*U*> exhibited beat phenomena and a high velocity. The resonance frequency was estimated at approximately *f* = 50 Hz. At *f* = 100 Hz, the velocity decreased slightly and did not exhibit beat phenomena as at *f* = 50 Hz. When the frequency was moved to *f* = 400 Hz, the velocity decreased substantially and did not exhibit pulsatile patterns. From the results, to obtain a consistent flow pattern of the reference fluid, it was confirmed that a lower frequency (*f* = 400 Hz) was much better than a higher frequency (*f* = 50–100 Hz).

Based on the averaged velocity (<*U*>) obtained with micro-PIV, the flow rate controlled by the micropump (*Q_mp_*) was then obtained as *Q_mp_* = *C_Q_* × *A_c_* × <*U*>. By inverting the slope (*Q_µPIV_*/*Q_sp_* = 0.8652) obtained at Hct = 1% (Figure 2B(a)), the correction factor (*C_Q_*) was calculated as 1.156. In addition, to represent the degree of pulsatile flow, instead of the commonly used pulsatile index (PI) (PI = [*Q_max_* − *Q_min_*]/*Q_ave_*, *Q_max_*: maximum flow rate, *Q_min_*: minimum flow rate, and *Q_ave_*: averaged flow rate), the coefficient of variance (COV) was suggested as COV = *Q_std_*/*Q_ave_* × 100 (%), where *Q_std_* was the standard deviation of the flow rate.

Figure 3C shows the variations in *Q_mp_* and COV with respect to *f* = 50, 100, 150, 200, 250, 300, 350, and 400 Hz. Here, the voltage was set to *v* = 150 au. *Q_mp_* decreases gradually when the frequency increases from *f* = 50 Hz. The COV decreases significantly at higher frequencies. The frequency was set as *f* = 400 Hz, where the COV had the minimum value. Furthermore, to select voltage as the other parameter, *Q_mp_* was obtained by varying the voltage from 100 au to 250 au. Figure 3D shows the variations in *Q_mp_* and COV with respect to *v* = 100, 150, 200, and 250 au. The frequency was set to *f* = 400 Hz. The results indicate that *Q_mp_* is linearly proportional to the voltage. The COV decreases substantially above *v* = 150 au. Based on these results, the voltage of the controller was fixed at *v* = 150 au. From the quantitative evaluations of the micropump, two control variables were selected, i.e., *f* = 400 Hz and *v* = 150 au, to guarantee a consistent flow rate of the reference fluid, even without a fluidic stabilization technique [43].

### 3.3. Quantitative Comparison between the Present Method and Previous Method for Measuring Biophysical Properties of Blood Sample

In the previous steps, the reference fluid and control variables of the micropump were selected for quantifying the flow rate of the reference fluid with consistency. It was necessary to validate the performance of the proposed method (i.e., micropump) by comparing it with the performance of a previous method (i.e., syringe pump). The previous method adopted a syringe pump with a constant flow rate of *Q* =1 mL/h. However, the proposed method used a micropump for supplying the reference fluid at a constant flow rate. The control variables (i.e., *f* = 400 Hz and *v* = 150 au) were set to operate the micropump to supply a reference fluid with RBCs (Hct = 1%). To validate the performance of the proposed method, the flow rate of the reference fluid was quantified by varying the junction pressure in a comparator. Regarding blood sample injections using both methods, after injecting blood samples (Hct = 50, RBCs suspended into 1× PBS) into a disposable syringe (~ 1 mL), the syringe was installed at the syringe pump. To vary the junction pressure in the comparator, the flow rate of the blood sample (*Q_b, sp_*) was increased from 0.5 mL/h to 5.5 mL/h at intervals of 0.5 mL/h.

**Figure 4:**
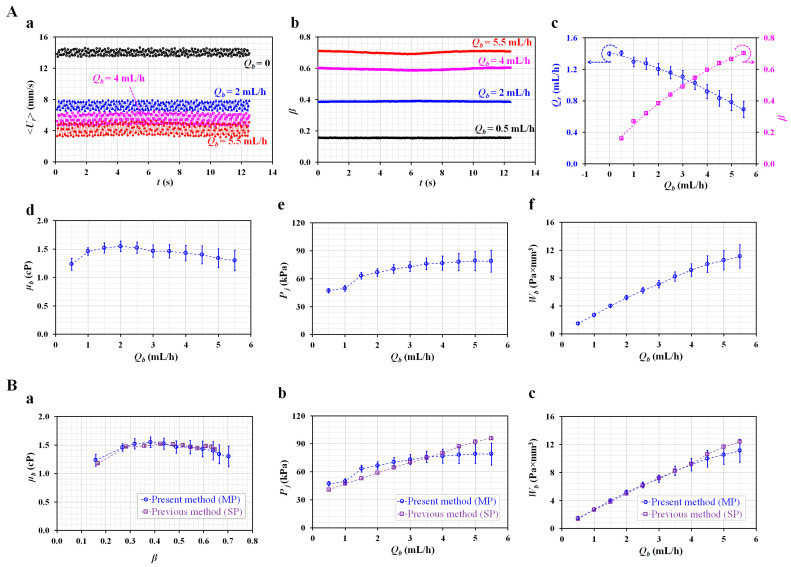
Quantitative evaluation of three biophysical properties (i.e., viscosity, junction pressure, and pressure-induced work) under a constant flow rate of the blood sample. (**A**) Quantitative evaluation of blood biophysical properties under constant blood flow set by a syringe pump. (**a**) Temporal variations of <*U_r_*> with respect to *Q_b_* = 0, 2, 4, and 5.5 mL/h. (**b**) Temporal variations of *β* with respect to *Q_b_*. (**c**) Variations of *Q_r_* and *β* with respect to *Q_b_*. (**d**) Variations of *µ_b_* with respect to *Q_b_*. (**e**) Variations of *P_j_* with respect to *Q_b_*. (***f***) Variations of *W_b_* with respect to *Q_b_*. (**B**) Quantitative comparison between the present method and previous method. (**a**) Comparison of *µ_b_* with respect to *β*. (**b**) Comparison of *P_j_* with respect to *Q_b_*. (**c**) Comparison of *W_b_* with respect to *Q_b_*.

As shown in Figure 4A, the biophysical properties of the blood obtained using the present method are summarized with respect to the constant flow rate of the blood sample. Figure 4A(a) shows the temporal variations in the averaged velocity of the reference fluid (<*U_r_*>) with respect to *Q_b_* = 0, 2, 4, and 5.5 mL/h. <*U_r_*> tends to decrease at a higher blood sample flow rate (i.e., high blood pressure in the comparator). The pulsatility of <*U_r_*> increases at higher *Q_b_* values. Figure 4A(b) shows the temporal variations in *β* with respect to *Q_b_*. The flow rate of the blood sample (*Q_b_*) contributes to increasing *β*. However, the pulsatility of *β* does not appear substantial, even at higher values of *Q_b_*. The averaged velocity (<*U_r_*>) was converted into the flow rate (*Q_r_*) using a simple formula (i.e., *Q_r_* = *C_Q_* × *A_c_* × <*U_r_*>). Both parameters (*Q_r_* and *β*) are represented as the mean ± standard deviation with respect to *Q_b_*. As shown in Figure 4A(c), *Q_r_* and *β* are represented with respect to *Q_b_*.

The flow rate of the reference fluid (*Q_r_*) gradually decreases with increasing *Q_b_*. The value of *β* tends to increase at higher *Q_b_* values. According to Equation (5), a higher value of β indicates that the junction pressure (*P_j_*) increases. The higher flow rate of the blood sample contributes to an increase in the junction pressure and causes a decrease in the flow rate of the reference fluid. A reciprocal relationship exists between *Q_r_* and *P_j_* (i.e., *Q_r_*–*P_j_*^−1^) [44].

Three biophysical properties (viscosity, junction pressure, and pressure-induced work) were then obtained, i.e., by adding *Q_r_* and *β* into Equation (8) for the blood viscosity (*µ_b_*), Equation (5) for the junction pressure (*P_j_*), and Equation (10) for the pressure-indexed work (*W_b_*). As the blood flow rate remained constant with the syringe pump, it was possible to obtain the blood viscosity under various flow rates of the blood sample. Figure 4A(d) shows the variations in µ_b_ with respect to *Q_b_*. Except at *Q_b_* = 0.5 mL/h, the blood viscosity remains constant with respect to *Q_b_* (i.e., *µ_b_* = 1.42 ± 0.1 cP, COV = 7%). The corresponding *β* of each flow rate was then obtained as 0.159 ± 0.003 (*Q_b_* = 0.5 mL/h) and 0.269 ± 0.004 (*Q_b_* =1 mL/h). According to a previous study [45], the equivalent diameter for each interface (i.e., $d_{e}=\sqrt{\frac{4\times \beta \times w\times h}{\pi }}$) was estimated as *d_e_* = 200 µm for *β* = 0.159 and *d_e_* = 260 µm for *β* = 0.269. It was inferred that the difference in the equivalent diameter (i.e., the effect of the cell-free layer) contributed to the varying blood viscosity in the comparator [35,46]. In addition, as *Q_r_* tended to decrease and exhibited large fluctuations at higher values of *Q_b_*, µ_b_ showed large scatters at higher values of *Q_b_*. To measure the blood viscosity with consistency, the interface was relocated within a specific range of 0.3 to 0.6 by adjusting the control variables (i.e., frequency and voltage). Figure 4A(e) shows the variations in *P_j_* with respect to *Q_b_*. As expected, *P_j_* gradually increases at higher values of *Q_b_*. As shown in Figure 4A(c), the scatter in *Q_r_* has an influence on those in *P_j_*, especially at higher values of *Q_b_*. Figure 4A(f) shows the variations in *W_b_* with respect to *Q_b_*. The results indicate that *Q_b_* substantially increases the pressure-induced work (*W_b_*). From the results obtained with the micropump, the three properties of the blood sample (i.e., viscosity, junction pressure, and pressure-induced work) were obtained consistently under constant flow rates for the blood sample.

**Figure A3:**
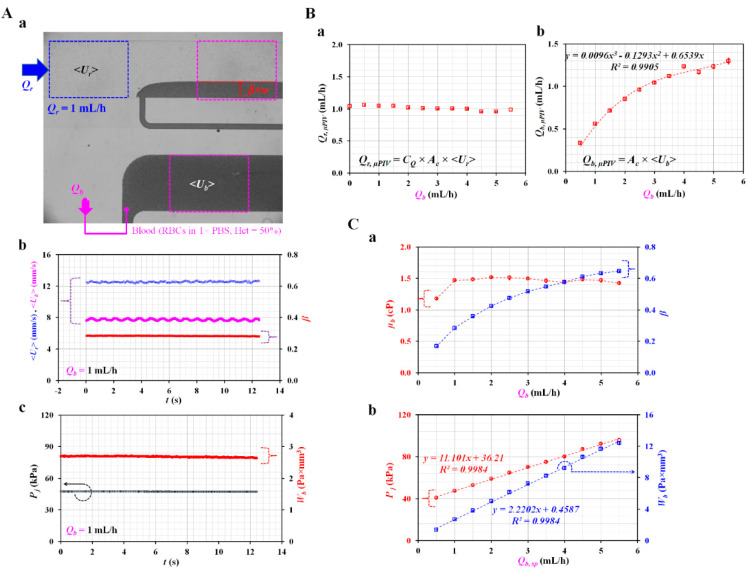
Quantification of three blood biophysical properties obtained by analyzing blood flows controlled with a syringe pump. (**A**) Quantification of junction pressure (*P_j_*) and pressure-induced work (*W_b_*) with three parameters. (**a**) Three parameters (i.e., <*U_r_*>, <*U_b_*>, and *β*) for estimating *µ_b_*, *P_j,_* and *W_b_* of blood flows. (**b**) Temporal variations of <*U_r_*>, <*U_b_*> and *β* over time. (**c**) Temporal variations of *P_j_* as well as *W_b_*. (**B**) Quantification of flow rates (*Q_r,µPIV_*, and *Q_b,µPIV_*) obtained by conducting micro-PIV. (**a**) Variations of *Q_r,µPIV_* with respect to *Q_b_*. (**b**) Variations of *Q_b,_µ_PIV_* with respect to *Q_b_*. (**C**) Variations of three blood properties with respect to the flow rate of the blood sample. (**a**) Variations of *µ_b_* and *β* with respect to *Q_b_*. (**b**) Variations of *P_j_* and *W_b_* with respect to *Q_b_*.

Second, to compare with the results obtained with the present method (i.e., the micropump), the previous method was adopted by replacing the micropump with a syringe pump, as shown in Figure A3A. The flow rate of the reference fluid was set at 1 mL/h (*Q_r_* = 1 mL/h). Based on the same protocols used in the present method, three parameters (<*U_r_*>, <*U_b_*>, and *β*) were obtained to estimate *µ_b_*, *P_j_*, and *W_b_*. Figure A3B(a) shows the variations in the flow rates (i.e., *Q_r,µPIV_*, and *Q_b,µPIV_*) as obtained with micro-PIV. *Q_r,µPIV_* remains unchanged with respect to *Q_b_*. The syringe pump was used to maintain the flow rate of the reference fluid. However, as shown in Figure 4A(c), the flow rate of the reference fluid controlled by the micropump was strongly influenced by the flow rate of the blood sample (i.e., the junction pressure in the comparator). As shown in Figure A3B(b), with respect to the flow rate of the blood sample controlled with the syringe pump, the flow rate obtained by micro-PIV (*Q_b,µPIV_*) exhibits a nonlinear relationship with the flow rate of the syringe pump (*Q_b_*). Based on a non-linear regression analysis, the variation in *Q_b,µPIV_* is best fitted as *Q_b,µPIV_* = 0.0096 *Q_b_*^3^ − 0.1293 *Q_b_*^2^ + 0.6539 *Q_b_* (*R*^2^ = 0.9905). According to a previous study [29], the flow rates of blood samples obtained by micro-PIV showed non-linear expressions with respect to hematocrit as well as given syringe flow rate. In addition, the RBC deformability and RBC aggregation contribute to varying flow rates (or velocity fields) obtained by micro-PIV. For this reason, micro-PIV has a limitation in obtaining accurate information on blood flows. Figure A3C shows the variations in three blood properties (i.e., *µ_b_*, *P_j_*, and *W_b_*) with respect to *Q_b_*. Above *Q_b_* =1 mL/h, the blood viscosity remains unchanged (i.e., *µ_b_* = 1.45 ± 0.09 cP, COV = 6.5%). With respect to *Q_b_* = 0.5 mL/h (i.e., *β* = 0.168 ± 0.001), the equivalent diameter is estimated as *d_e_* = 205 µm. Furthermore, as the linear regression analysis provides a higher value of the regression coefficient (*R^2^* = 0.9984), it can be confirmed that *P_j_* and *W_b_* have linear relationships with respect to *Q_b_*.

As shown in Figure 4B, the biophysical properties (*µ_b_*, *P_j_*, and *W_b_*) obtained by both methods overlap with respect to *β* or *Q_b_*. Figure 4B(a) shows *µ_b_* with respect to *β*. Both methods show consistent trends in regard to blood viscosity. However, the present method (i.e., micropump) exhibits slight fluctuations relative to the previous method (i.e., syringe pump). Figure 4B(b) shows a comparison of *P_j_* with respect to *Q_b_*. The syringe pump (i.e., the previous method) maintains a consistent flow rate of the reference fluid, and the junction pressure tends to increase linearly with respect to *Q_b_*. However, the flow rate of the reference fluid, as controlled by the micropump, varies substantially according to the flow rate of the blood sample (*Q_b_*). Thus, at higher flow rates of the blood sample (i.e., *Q_b_* > 3.5 mL/h), the present method underestimates the junction pressure relative to the previous method. Figure 4B(c) shows a comparison of *W_b_* with respect to *Q_b_*. Both methods yield consistent results, except for the higher flow rate of the blood sample (i.e., *Q_b_* > 4.5 mL/h). From a quantitative comparison, although a syringe pump (i.e., the previous method) was replaced by a micropump (i.e., the present method) to supply the reference fluid into the microfluidic channels, the three biophysical properties of the blood sample were obtained with sufficient consistency.

**Figure 5:**
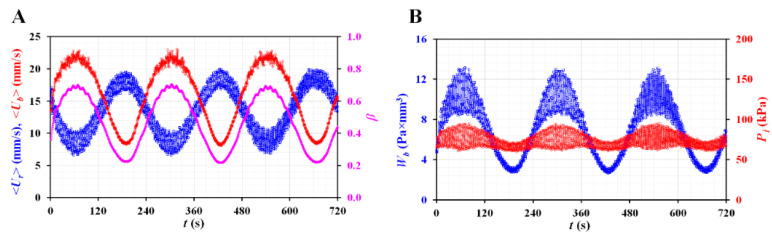
Quantitative evaluations of two biophysical properties (i.e., junction pressure and pressure-induced work) under periodic blood flow. Using a syringe pump, blood flow rate (*Q_b_*) was supplied as *Q_b_* (*t*) = 3 + 2 sin (2 × π × *t*/240) mL/h. (**A**) Variations of <*U_r_*>, <*U_b_*>, and *β* with an elapse of time. (**B**) Temporal variations of *P_j_*, and *W_b_*.

Finally, instead of a constant flow rate for the blood sample, the blood sample was injected as a peristaltic flow (i.e., *Q_b_* [*t*] = 3 + 2 sin [2 × π × *t*/240 mL/h]). The reference fluid was supplied using the micropump (i.e., *f* = 400 Hz, *v* = 150 au). Figure 5A shows the variations in <*U_r_*>, <*U_b_*>, and (*β*) with time. Depending on the periodic flow rate of the blood sample, <*U_r_*> represents the periodic patterns; <*U_r_*> has a reciprocal relationship with <*U_b_*> (i.e., 180° out of phase) and *β* varies in phase when compared with <*U_b_*>. Considering that the micro-PIV did not provide an accurate flow rate of the blood sample, it was impossible to obtain the blood viscosity over time. Thus, two biophysical properties of the blood sample (i.e., junction pressure and pressure-induced work) were obtained by analyzing *Q_r_* and *β*. Figure 5B shows the temporal variations in *P_j_* and *W_b_*. Both properties exhibit periodic patterns and vary in phase with respect to <*U_b_*> (or *β*).

From the experimental comparisons, under a constant or periodic flow rate of the blood sample, the syringe pump can be replaced with a micropump for supplying the reference fluid. Moreover, the present method has the ability to obtain the biophysical properties of blood samples with sufficient accuracy.

### 3.4. Quantitative Evaluations of Biophysical Properties of Blood Sample

Based on the present method (i.e., micropump), the biophysical properties of the blood samples were obtained by quantifying the blood flows in the comparator. According to previous studies [47], dextran (with a high molecular weight) was used to change the RBC aggregation [14,15,18], viscosity [30], and velocity profiles [48]. Thus, to prepare blood samples with substantially different biophysical properties, different concentrations of dextran solution were added to normal RBCs according to our previous studies [32,49]. Several blood samples (Hct = 50%) were prepared by adding normal RBCs to dextran solutions of specific concentrations ranging from 0 to 40 mg/mL at intervals of 10 mg/mL. A reference fluid with a specific RBC volume fraction (Hct = 1%) was supplied to the RC with a micropump (i.e., *f* = 400 Hz and *v* = 150 au). Simultaneously, by setting a syringe pump in an on-off fashion (i.e., *Q_b_* = 1 mL/h and on-off period = 240 s), the blood samples were supplied to the BC.

**Figure 6:**
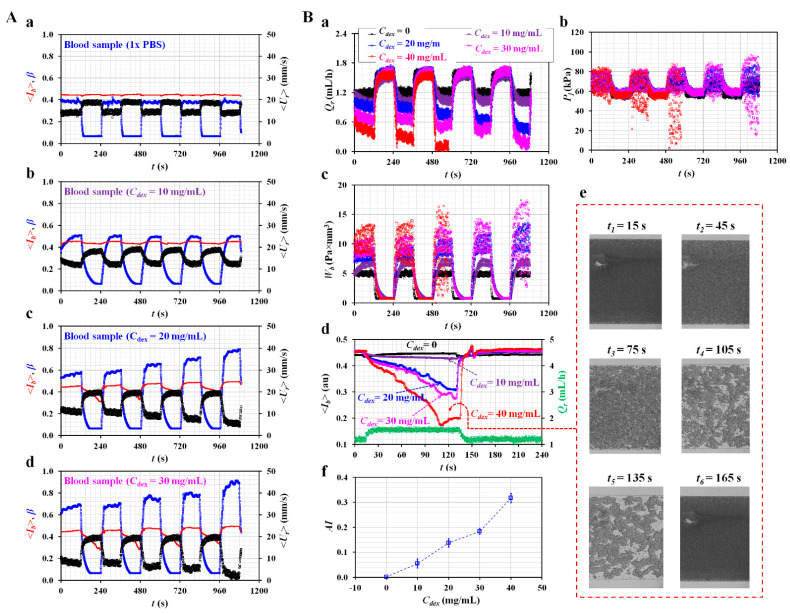
Quantitative evaluations of three biophysical properties of blood sample. (**A**) Temporal variations of <*U_r_*>, <*I_b_*> and *β* with respect to concentration of dextran solution (*C_dex_*) ([**a**] *C_dex_* = 0, [**b**] *C_dex_* = 10 mg/mL, [**c**] *C_dex_* = 20 mg/mL, and [**d**] *C_dex_* = 30 mg/mL). (**B**) Contributions of dextran solution to biophysical properties of blood sample (i.e., junction pressure, pressure-induced work, and RBC aggregation). (**a**) Temporal variations of *Q_r_* with respect to *C_dex_*. (**b**) Temporal variations of *P_j_* with respect to *C_dex_*. (**c**) Temporal variations of *W_b_* with respect to *C_dex_*. (**d**) Temporal variations of <*I_b_*> with respect to *C_dex_*. (**e**) Microscopic images of blood sample (*C_dex_* = 40 mg/mL) captured at a specific time (*t*) (*t* = 15, 45, 75, 105, 135, and 165 s). (**f**) Variations of RBC aggregation index (*AI*) with respect to *C_dex_*.

Figure 6A depicts the temporal variations of <*U_r_*>, <*I_b_*> and *β* with respect to concentrations of the dextran solution (*C_dex_*) ([a] *C_dex_* = 0, [b] *C_dex_* = 10 mg/mL, [c] *C_dex_* = 20 mg/mL, and [d] *C_dex_* = 30 mg/mL). <*U_r_*> shows periodic up-and-down variations, owing to the periodic on-off flow of the blood sample. The dextran solution contributes substantially to increasing the transient time (i.e., characteristic time).

The concentration of <*U_r_*> decreased significantly at higher concentrations of the dextran solution. For example, for *C_dex_* = 30 mg/mL, the reference fluid flow stopped after *t* = 960 s. When the blood sample was supplied to the BC, *β* increased considerably at higher dextran solution concentrations. During the turn-off period of the blood flow, the dextran solution significantly changed the transient profile of *β*. In addition, the change in <*I_b_*> increased substantially at higher dextran concentrations. Using the temporal variations in <*U_r_*>, <*I_b_*>, and β, as shown in Figure 6A, the flow rate of the reference fluid (*Q_r_*), junction pressure (*P_j_*), pressure-induced work (*W_b_*), and RBC aggregation index (*AI*) [15] were obtained with respect to *C_dex_*. Figure 6B(a) shows the temporal variations in *Q_r_* with respect to *C_dex_*. During the turn-on period of blood flow, *Q_r_* decreases significantly at higher concentrations of the dextran solution. In addition, it varies significantly with increasing time. With respect to *C_dex_* = 40 mg/mL, the reference fluid flow stops after *t* = 480 s. Figure 6B(b) shows the temporal variations of *P_j_* with respect to *C_dex_*. With respect to *C_dex_* = 30 and 40 mg/mL, there are large fluctuations during the turn-on period of the blood flow. Figure 6B(c) shows the temporal variations in *W_b_* with respect to *C_dex_*. *W_b_* increases significantly at higher concentrations of the dextran solution. With respect to *C_dex_* = 30 and 40 mg/mL, W_b_ exhibits large fluctuations which increase significantly with time. Considering that the micro-PIV did not provide accurate information regarding the blood, this study did not attempt to obtain the blood viscosity of blood samples as a function of time. However, according to Equations (5), (8), and (10), the interface (*β*), which is influenced by the blood viscosity, contributes substantially to the changes in *P_j_* and *W_b_*. For this reason, these two biophysical properties (i.e., *P_j_* and *W_b_*) can be employed to screen for changes in blood samples with sufficient consistency.

**Figure A4:**
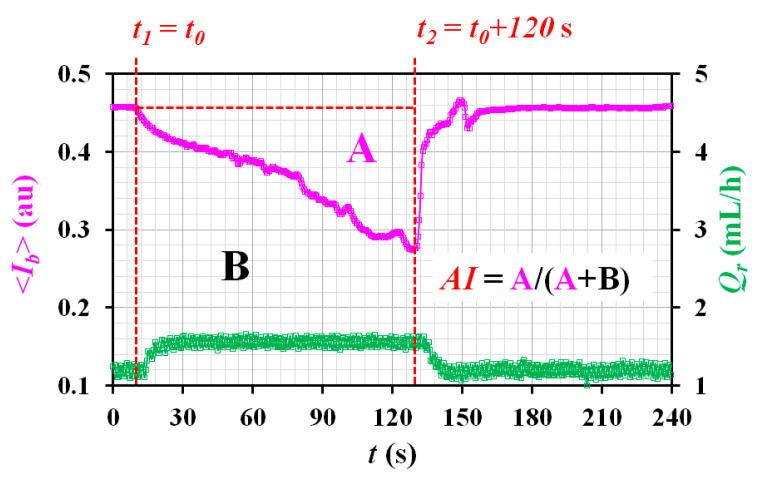
Quantification of RBC aggregation index (*AI*). Based on temporal variations of <*I_b_*>, A and B were calculated for Δ*t* = *t*_2_ − *t*_1_; the *AI* was then obtained as *AI* = A/(A + B).

However, as the syringe pump supplied the blood samples in an on-off fashion, the RBCs aggregated and disaggregated periodically. During the turn-off period, the RBC aggregation substantially changed the microscopic image intensity over time. Thus, based on temporal variations of <*I_b_*>, the variations in the RBC aggregation were quantified using the well-known formula of the RBC aggregation index (*AI*). Figure 6B(d) shows the temporal variations of <*I_b_*> with respect to *C_dex_* for a single on-off period of blood flow, where *Q_r_* is the flow rate of the reference fluid (*C_dex_* = 0). With respect to *C_dex_* = 40 mg/mL, <*I_b_*> saturates after Δ*t* = 96 s. Except for *C_dex_* = 40 mg/mL, <*I_b_*> gradually increases over time. The results indicate that the dextran solution contributes to changing <*I_b_*> largely with the elapse of time. Figure 6B(e) shows microscopic images of blood samples (*C_dex_* = 40 mg/mL) captured at specific times (*t*) (*t* = 15, 45, 75, 105, 135, and 165 s). With an increase in time, RBC aggregation and increased RBC-free spaces are clearly observed. It is estimated that the RBC-free space contributes considerably to increasing <*I_b_*>. To obtain quantitative information on the RBC aggregation, based on previous studies [15], the RBC aggregation index (*AI*) was defined as *AI* = A/(A + B). As shown in Figure A4, after turning off the syringe pump, A and B were obtained by analyzing <*I_b_*> for Δ*t* = *t_2_* − *t_1_* = 120 s (i.e., *t_1_* = *t_0_* and *t_2_* = *t_0_* + 120 s). Figure 6B(f) shows the variations in the *AI* with respect to *C_dex_*. The AI increases substantially at higher concentrations of the dextran solution. According to a previous method [15], a straight or large circular channel was employed to quantify the contribution of the dextran solution (maximum concentration of the dextran solution: 15–25 mg/mL). The present method adopted a unique blood channel consisting of three channels (i.e., narrow channel—big channel—narrow channel) connected in series. When the syringe pumps were turned off, the blood flow stopped immediately. Additionally, the reference fluid did not invade the RC. Owing to the unique structure of the RC, the present method could provide consistent variations in the *AI*, especially at higher concentrations of the dextran solution (40 mg/mL). From the results, even while turning off the blood flow, the present method was able to quantify the RBC AI with consistency by analyzing the temporal variations of microscopic image intensity.

From the experimental demonstration, the present method was validated as an effective method for quantifying the physical properties of blood samples by analyzing blood flows in a comparator. As a limitation, the present method was demonstrated with bulk-sized equipment, including a microscope and high-speed camera. In addition, the flow rate of the blood sample was specifically set to obtain the blood viscosity using a syringe pump. To resolve the issues regarding a more portable rheometer [25], the present method will be improved in the near future by adapting a portable imaging acquisition system [3,50,51,52,53,54], and by adding passive pumps [55].

## 4. Conclusions

In this study, we proposed the quantification of blood biophysical properties based on junction pressure sensing with a micropump and microfluidic comparator. The previous bulk-sized high-precision syringe pump was replaced with a portable and cheap micropump. The micropump was adopted to supply reference fluid. Flow rate (or velocity profile) varied with the junction pressure in the microfluidic comparator. The flow rate of the reference fluid was quantified continuously with the micro-PIV technique. Here, a small fraction of RBCs (Hct = 1%) as fluid tracers was added to the reference fluid. To avoid RBCs as a fluid tracer in the reference fluid stream, a specific concentration of glycerin solution (40%) was selected as the reference fluid. It was then possible to obtain the flow rate of the reference fluid with sufficient consistency. Analytical expressions for three biophysical properties of blood samples were derived using a discrete fluidic circuit model. The accuracy of the mathematical model was validated when compared with experimental results. The present method was employed to quantify the biophysical properties of blood samples, where the flow rate of the blood sample was specified under constant, sinusoidal, and periodic on-off patterns. From the quantitative comparison study, the present method (i.e., micropump) provided comparable results when compared to the previous method (i.e., macroscopic pump). In conclusion, the present method could be considered as an effective method for quantifying the physical properties of blood samples flowing in the microfluidic comparator where the reference fluid was supplied with a cheap and portable micropump.
